# Empagliflozin: a potential anticancer drug

**DOI:** 10.1007/s12672-023-00719-x

**Published:** 2023-07-12

**Authors:** Wenwen Wu, Yanyan Wang, Jun Xie, Shaohua Fan

**Affiliations:** 1 School of Life Sciences, Jiangsu Normal University, Xuzhou, Jiangsu 221116 China; 2 Department of Ultrasonic Medicine, The First People’s Hospital of Xuzhou, Xuzhou Municipal Hospital Affiliated to Xuzhou Medical University, Xuzhou, Jiangsu 221000 China

**Keywords:** Empagliflozin, SGLT2 inhibitor, Anticancer, Cancer therapy

## Abstract

Empagliflozin, a sodium-glucose cotransporter 2 (SGLT2) inhibitor, is a highly effective and well-tolerated antidiabetic drug. In addition to hypoglycemic effects, empagliflozin has many other effects, such as being hypotensive and cardioprotective. It also has anti-inflammatory and antioxidative stress effects in diabetic nephropathy. Several studies have shown that empagliflozin has anticancer effects. SGLT2 is expressed in a variety of cancer cell lines. The SGLT2 inhibitor empagliflozin has significant inhibitory effects on certain types of tumor cells, such as inhibition of proliferation, migration and induction of apoptosis. In conclusion, empagliflozin has promising applications in cancer therapy as a drug for the treatment of diabetes and heart failure. This article provides a brief review of the anticancer effects of empagliflozin.

## Introduction

Diabetes is a gradually growing chronic disease that is difficult to cure, with type 2 diabetes (T2D) accounting for more than 90% of adult onset diabetes [1]. Empagliflozin is a sodium-glucose cotransporter 2 inhibitor that has been approved for the treatment of T2D in adults in the EU, US, and Japan due to its high selectivity [2]. The pathogenesis of T2D includes insulin resistance or hyposecretion. Glucose in the body enters the kidneys and is reabsorbed into the blood via SGLT2 protein in the proximal renal gyral tubule [3]. Empagliflozin can lower blood glucose levels by blocking SGLT2 cotransport protein in the proximal renal gyral tubule, promoting urinary glucose excretion, and preventing glucose reabsorption [4], thus lowering blood glucose levels. The most important feature of empagliflozin is that it prevents the reabsorption of glucose by the kidneys and controls blood glucose compared to other drugs used to treat diabetes and is a hypoglycemic drug that does not depend on insulin action with no hypoglycemia risk. Therefore, it can be used as monotherapy or in combination with other drugs to treat T2D [5].

Empagliflozin has many other effects in addition to its hypoglycemic effect. This drug has been reported to exhibit renoprotective potential in diabetic nephropathy, depending in part on the inhibition of epithelial-mesenchymal transition (EMT) and abnormal glycolysis in renal tubular cells [6]. It may also have a preventive effect on cardiovascular disease by ameliorating metabolic abnormalities and hemodynamic abnormalities [5]. Furthermore, studies have demonstrated that empagliflozin can decrease oxidative stress and inflammatory markers in the lungs of mice, effectively preventing pulmonary fibrosis [7]. Additionally, in diabetic rats, it demonstrates antioxidant and anti-inflammatory properties in the kidneys [8]. Ojima et al. [9] found through in vivo experiments that empagliflozin reduced renal inflammation and oxidative stress through the inhibition of the AGE/RAGE/NF-κB axis, thereby reducing damage to renal tubular cells. Further analysis showed that this was mainly associated with empagliflozin reducing diabetes-associated renal HMGB1 protein levels [8]. Wu et al. [10] further demonstrated that the combination of this drug with ursolic acid can effectively suppress the TGF-β/SMAD/MAPK signaling pathway, leading to a decrease in renal inflammation, oxidative stress, and renal fibrosis. In addition, Iannantuoni et al. [11] found that empagliflozin has the potential to improve inflammation in patients with T2D due to the promotion of the antioxidant response of leukocytes. They followed 15 patients with T2D taking certain doses of empagliflozin and observed weight loss, reduced glucose levels, reduced superoxide in leukocytes, reduced pro-inflammatory markers, and increased anti-inflammatory parameters [11]. This provides strong evidence for the antioxidant and anti-inflammatory properties of empagliflozin, particularly in the prevention of cardiovascular disease [12, 13].

SGLT2 is expressed in a variety of tumor cells, such as pancreatic tumors, prostate tumors, and glioblastoma [14]. In a study by Shoda et al., it was confirmed that glioma cells utilize SGLT2 for glucose uptake and that the SGLT2 inhibitor canagliflozin inhibits glucose uptake by these cells [15]. Another study by Kaji et al. demonstrated that canagliflozin inhibits the proliferation of Huh7 and HepG2 cells by suppressing glucose uptake, lactate production, and intracellular ATP production [16]. While Empagliflozin, as an SGLT2 inhibitor, has also been reported to have inhibitory effects on cancer cells, such as cervical cancer [17], breast cancer [18], hepatocellular carcinoma(HCC) [19], and lung cancer [2]. One of the possible molecular mechanism is that it inhibits the reabsorption of glucose by the renal tubules, and the glucose required for the growth and metabolism of tumor cells is reduced [20], inhibiting the growth and proliferation of tumor cells. However, the specific mechanism of action needs to be further explored. Thus, empagliflozin is not only able to lower blood sugar but also a potential anticancer drug [17, 21]. This review summarizes the oncological studies related to empagliflozin, whose anticancer effects may become a breakthrough in cancer therapy.

## Current status of cancer research

### Current status of cancer

Cancer has long been a medical conundrum worldwide, characterized not only by a high mortality rate but also by the terrible suffering of patients at an advanced stage. By the end of 2022, there could be approximately 4.82 million new cancer cases and 3.21 million cancer deaths in China [22]. Presently, cancer treatments include radiotherapy, chemotherapy, and surgical resection. Surgical resection remains the primary form of treatment for solid tumors in clinical practice. However, precise tumor removal can be facilitated by multimodal imaging-based guided surgical resection [23], which can be followed by local radiation therapy and oral chemotherapeutic agents to maximize patient survival. Unfortunately, some tumors have a high risk of recurrence due to their extreme metastatic ability and infiltrative nature. Radiation therapy is an excellent adjunct to localized tumor treatments, as it uses high-energy ionizing radiation to induce apoptosis by generating ROS in tumor cells and damaging their DNA [24]. Nevertheless, radiation alone is less effective due to the hypoxic microenvironment [25]. Nanoparticles have shown considerable promise in tumor radiation therapy, and their combination has the potential to improve the tumor’s lack of oxygen [26] and enhance the therapeutic effect. Wang et al. developed pH-responsive functionalized nano-andrographic hydrogels coupled with 6-aminonicotinamide to inhibit the pentose phosphate pathway’s metabolism, thereby reducing NADPH production, inhibiting the conversion of oxidized glutathione to reduced glutathione, and enhancing sustained ROS production to increase radiotherapy sensitivity [27]. Furthermore, the incorporation of highly effective and specific anticancer drugs as radiosensitizers in combination with chemotherapy is expected to produce more effective therapeutic outcomes.

Recent technological advancements in medical research suggest that immunotherapy will play an important role in cancer treatment [28]. Immunotherapy is an emerging cancer treatment that has undergone clinical testing and has been successfully applied to different types of human tumors. It functions by stimulating the immune system to recognize and destroy cancer cells [29]. Notably, the PD-1/PD-L1 signaling pathway has been linked to tumor cells’ immune escape [30], and blocking this pathway by inhibiting *PD-L1* expression can help reactivate the T-cell immune response. Researchers such as Tang et al. have investigated the use of hollow Prussian blue nanoparticles encapsulating lactate oxidase and PD-L1 siRNA [31], while Song et al. have utilized carboxymethyl chitosan to target the delivery of doxorubicin and PD-L1 siRNA to tumor sites, leading to enhanced intra-tumor immunogenic cell death (ICD) effect and suppression of *PD-L1* expression [32]. Despite being a hot spot of clinical immunology research, PD-1/PD-L1 therapy has its limitations, and the detailed mechanism of action requires further exploration to alleviate toxic side effects and drug resistance and improve its therapeutic role in clinical practice.

### The current challenges with the existing anticancer therapies

In recent years, with the advancement of cancer treatments, systemic chemotherapy has emerged as an important tool in cancer treatment. Traditional chemotherapy drugs excel at killing cancer cells, but not all of them. As a result, the remaining cancer cells are able to metastasize and lesions may persist. Moreover, resistance to chemotherapeutic drugs has further limited the use of chemotherapy in clinical practice [33]. Combination therapies with other treatments are being explored as a potential solution to overcome resistance. Studies have shown that targeted therapies are capable of regulating the growth cycle of cancer cells and inducing apoptosis by identifying specific targets on the cancer cell surface [34]. In contrast, combining chemotherapy with targeted therapy can enable the delivery of chemotherapeutic drugs precisely to the tumor site, while encapsulation of nanocarriers can enhance the biosafety of chemotherapeutic drugs circulating in vivo. Though targeted therapy has proven effective in treating cancer, it works by acting on specific biomarkers in the development of cancer [34]. Hence, this mode of treatment is advantageous for a particular group of patients, and it is essential to identify the exact targets before commencing targeted therapy. A significant hurdle in targeted therapies is the pressing requirement to identify molecular targeting ligands and specific targets to precisely deliver nanocarriers with chemotherapeutic drugs [35]. Radiation therapy is frequently used clinically in combination with chemotherapy, but conventional radiation therapy can alter the tumor microenvironment and cause inflammation [36] with a killing effect on the normal tissue cells surrounding the tumor, thereby limiting its clinical application. While immunotherapy continues to show promise in the field of cancer research, it is not without clinical limitations. Despite the successes of some clinical drugs targeting immunotherapy in long-term treatment, drug resistance frequently occurs, and resistance mechanisms are complex [37, 38], making present clinical solutions suboptimal. Thus, comprehensively exploring and elucidating the mechanisms of drug resistance remains a major challenge for current cancer treatment. Optimizing chemotherapy drugs and developing effective, safe, and precise cancer therapies remain necessary to achieving successful cancer therapy in the future.

### Current status of antidiabetic agents in cancer research

Recently, researchers have studied several diabetes drugs and found them to have potential anticancer activity. Metformin is a first-line therapeutic drug used to treat T2D, and its antitumor effects have been demonstrated in several types of cancer [39–42]. Researchers have found that metformin reduces cancer risk in patients with T2D by activating the adenosine monophosphate (AMP)-activated protein kinase (AMPK) signaling pathway [43, 44]. Glitazone is also a common drug on the market for the treatment of diabetes. Glitazone can exert its antitumor activity by activating peroxisome proliferator-activated receptor gamma (PPARγ), mitogen-activated protein kinase (MAPK), inflammatory pathways, and transforming growth factor beta (TGFβ) [45]. Kuang et al. have demonstrated the inhibitory effects of dapagliflozin, a hypoglycemic drug, on renal cell carcinoma by inducing G1 phase arrest in Caki-1 cells. Furthermore, the apoptosis rate was found to be 1.89-fold higher in cells treated with dapagliflozin compared to the control group [46]. Similarly, canagliflozin, another hypoglycemic drug, has been shown to inhibit oxidative stress in rat breast cancer cells. This inhibition is achieved through a multi-pronged approach involving the enhancement of tumor suppressor gene *BRCA-1* expression, mTOR inflammatory pathway inhibition, and suppression of the expression of *NLRP3*, *GSDMD*, *NF-κB*, and *IL-1β* ultimately [47]. Empagliflozin is also a drug for the treatment of T2D and has been shown to inhibit the growth of a variety of cancer cells (Table 1). The drugs mentioned above for the treatment of diabetes have shown some inhibitory effects on certain types of cancer. The combination of these drugs with therapies such as radiotherapy or photothermal therapy is expected to produce a synergistic therapeutic effect.

**Table 1:** Potential therapeutic effects of empagliflozin on multiple cancers

Cancer type	Drug	In vitro/In vivo	Results	References
Cervical cancer	Empagliflozin	In vitro In vivo	Activated the AMPK signaling pathway, inhibited the expression of *FOXA1* and *SHH* to inhibit the proliferation, migration and induction of apoptosis in cervical cancer cells	[17]
Breast cancer	Empagliflozin	In vitro	Inhibited the expression of *MDR1* and increased cell sensitivity to doxorubicin	[18]
			Downregulated the expression of *mTOR*, *Bcl-2*, and *JNK* and promoted cell apoptosis	
			Upregulated the expression of *p21* and promoted cell apoptosis	
Hepatocellular Carcinoma	Empagliflozin and metformin	In vivo	Activated AMPK and inhibited the expression of *mTOR*, leading to NF-κB inactivation and inhibition of hepatocellular carcinoma growth	[19]
			Inhibited t0he expression of *MAPKs*, *p38* and *ERK1/2*	
Lung cancer	Empagliflozin	In vitro	Promoted cell apoptosis	[2, 70]
		In vivo	Exhibited cytotoxicity against A549 cells and improved bioavailability	

## Empagliflozin and cancer

### Empagliflozin and cervical cancer

**Fig. 1:**
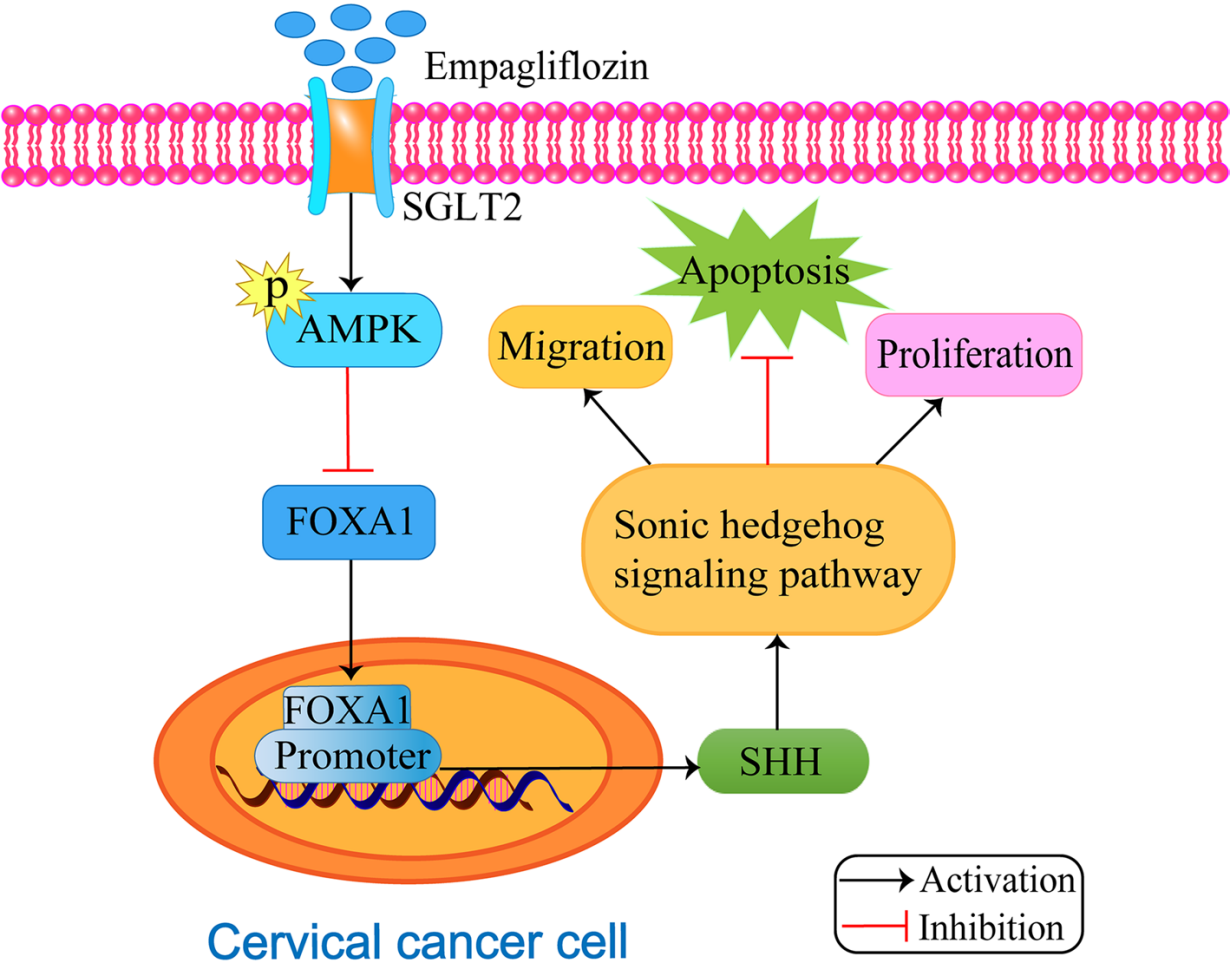
The mechanism of action of empagliflozin in cervical cancer. Effects of empagliflozin on proliferation, migration and apoptosis of cervical cancer cells and possible molecular mechanisms

Cervical cancer is the second most common malignancy among women worldwide, and human papillomavirus (HPV) infection is the main cause of cervical cancer. Studies have shown that early diagnosis and HPV vaccine application can prevent the occurrence of cervical cancer, but the low HPV vaccination rates make cervical cancer a serious threat to women’s health in developing countries [48]. Although studies have shown that high-risk human papillomavirus (hrHPV) can be detected in almost all types of HPV-induced cervical cancer [49], the pathogenesis of this disease remains poorly understood. In their study, Xie et al. discovered that empagliflozin can suppress the development and progression of cervical cancer by activating the adenosine monophosphate (AMP)-activated protein kinase (AMPK) signaling pathway and downregulating the expression of forkhead Box A1 (*FOXA1*) and sonic hedgehog (SHH) (Fig. 1) [17]. Empagliflozin also demonstrated antitumor activity in vitro, as it enhanced caspase 3 cleavage activity and *Bax*/*Bcl-2* expression [17]. In vivo experiments involved subcutaneously inoculating HeLa cells, a cervical cancer cell line, treated with cyclophosphamide (CTX) or empagliflozin into nude mice, with CTX treated cells serving as a positive control. The researchers injected mice with a 50 µM empagliflozin solution, and the tumors were observed to be significantly smaller in size [17]. The authors suggest that the antitumor effects of empagliflozin are due to its ability to increase levels of AMPK phosphorylation by blocking glucose uptake while inhibiting *FOXA1* expression [17]. Unlike CTX, which has toxic side effects, empagliflozin has a higher safety profile with fewer side effects. Future research should focus on understanding the precise mechanism of action of empagliflozin in cervical cancer treatment and the development of a new drug delivery system to resolve its poor water solubility for targeted drug delivery to the tumor site. This could help maximize the potential of empagliflozin as a treatment option for cervical cancer patients and open up new avenues for clinical treatment of the disease.

### Empagliflozin and breast cancer

**Fig. 2:**
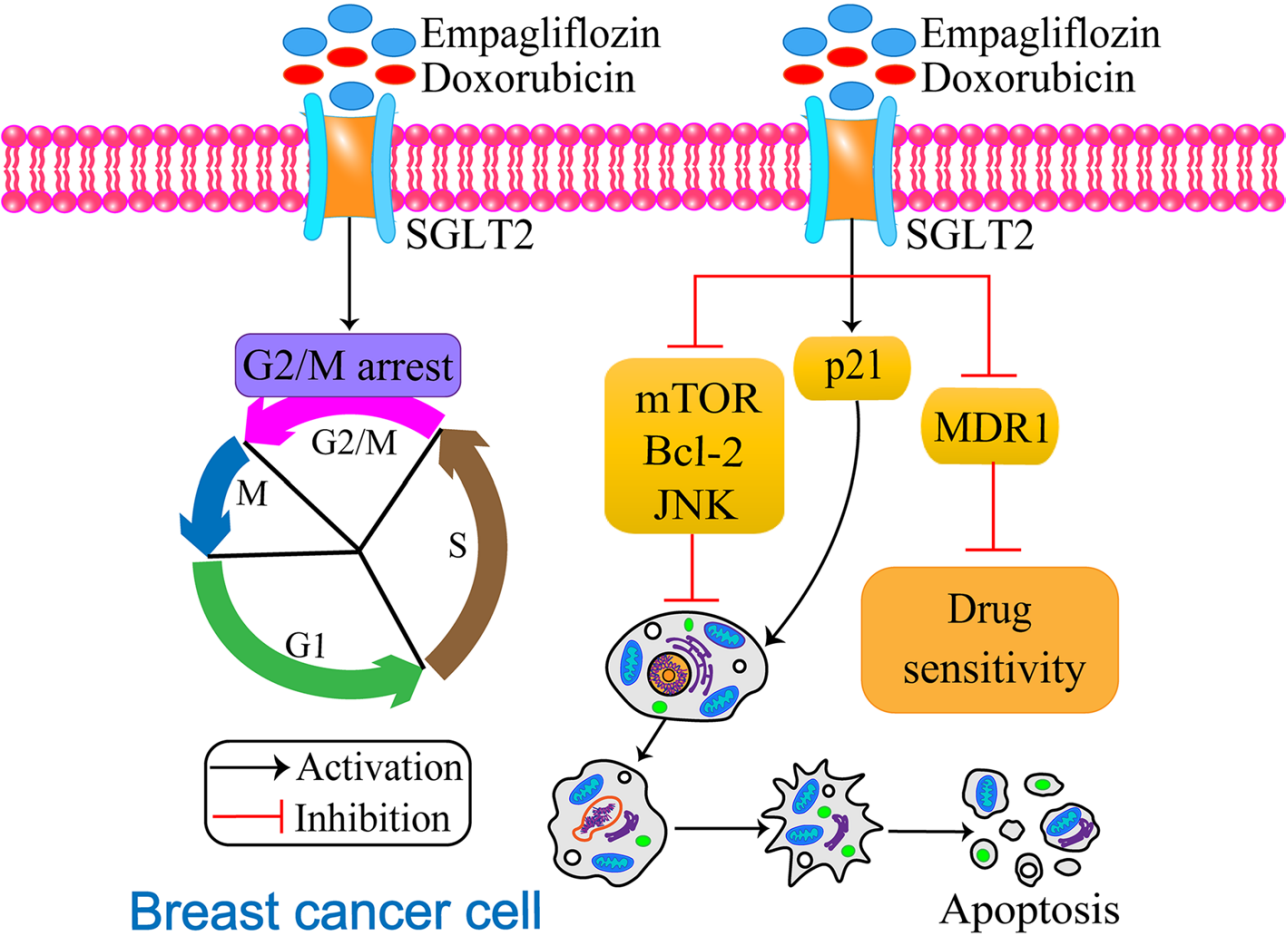
The mechanism of action of empagliflozin and doxorubicin in synergistic treatment of breast cancer. The combination treatment significantly inhibits the expression of proliferative genes *mTOR*, *JNK*, and *Bcl-2*, and upregulates the expression of anti-proliferative gene *p21*. Additionally, it decreases the expression of *MDR1*, thereby increasing the sensitivity of MDA-MB-231 cells to doxorubicin

Triple-negative breast cancer (TNBC) is a highly aggressive subtype of breast cancer. It accounts for 10–20% of all breast cancers [50]. TNBC is highly drug-resistant, and common chemotherapeutic drugs do not work well against TNBC. There has been no effective treatment for TNBC, but some researchers have found that the phosphoinositide 3-kinase (PI3K)/protein kinase B (AKT)/mechanistic target of rapamycin (mTOR) pathway may be a potential target for the treatment of TNBC [51]. Based on this study, researchers found that inhibitors of AKT improved the prognosis of a subset of patients with metastatic TNBC when combined with the first-line chemotherapy drug paclitaxel [52]. Further exploration of the combination of the two drugs is underway. Doxorubicin (DOX) is an anthracycline-based chemotherapeutic drug used in the treatment of many types of cancer [53]. DOX is the drug of choice for the treatment of TNBC, but many cancer patients develop resistance to DOX [54], so there is a need to investigate a drug that resensitizes tumor cells to DOX for the combination treatment of TNBC. Empagliflozin resensitizes tumor cells to DOX [18], improves patient resistance to DOX and has cardioprotective effects. In contrast, empagliflozin has recently been shown to have the ability to promote apoptosis in the breast cancer cell line MCF-7 [2]. Eliaa et al. [18] combined empagliflozin with DOX to synergistically inhibit the survival of TNBC cells by interfering with the mTOR pathway and inhibiting calmodulin (Fig. 2). To explore the molecular mechanism of the combined action of empagliflozin and DOX, the authors performed a molecular docking study and found that empagliflozin blocked calmodulin receptors by binding to a cocrystallized inhibitor of DOX [55]. This suggests that empagliflozin acts as a calmodulin receptor antagonist with a chemosensitizing effect on DOX [18] and demonstrates that empagliflozin has a chemosensitizing effect on DOX by inhibiting the expression of the *MDR1* gene [18]. In a recent study, it was found that empagliflozin, when combined with doxorubicin, can significantly inhibit the expression of the proliferative genes *mTOR*, *JNK*, and *Bcl-2* while upregulating the expression of the anti-proliferative gene *p21* [18]. While the effects of empagliflozin on cytotoxicity were not significant, the combination of empagliflozin and doxorubicin treatment resulted in a reduced number of cells in G2/M and a 1.42-fold increase in cell cycle arrest compared to doxorubicin alone treated group [18]. This suggests that empagliflozin combined with doxorubicin treatment can arrest the cell cycle in G2/M phase. Moreover, the IC_50_ value for the combination of doxorubicin and empagliflozin was 1.70 µM, while the IC_50_ value for doxorubicin treatment alone on breast cancer cells was 1.23 µM, indicating that empagliflozin can increase sensitivity to doxorubicin [18]. Empagliflozin is mainly used as an adjuvant to reduce the toxicity of doxorubicin to cells and the heart, improve patients’ resistance to doxorubicin, and have a cardioprotective effect [18]. In addition, empagliflozin has been shown to inhibit calmodulin-dependent kinase II activity, as confirmed by the study of Mustroph et al. [56], which may contribute to its cardioprotective effects. Nalla et al. demonstrated that empagliflozin-induced miR-128-3p inhibits SP1 and PKM2 activity in hypoxic breast cancer cells, promotes CD44+/CD24 + differentiated cells, and reduces metastasis of breast cancer cells [57]. Empagliflozin has shown promise as a potential anticancer drug when used in combination with other clinical chemotherapeutic agents for the treatment of breast cancer. Further research into the synergistic effects of empagliflozin with conventional cancer therapy or other antidiabetic drugs for breast cancer is needed to support its observed in vitro and in vivo anticancer effects in this patient population.

### Empagliflozin and hepatocellular carcinoma

**Fig. 3:**
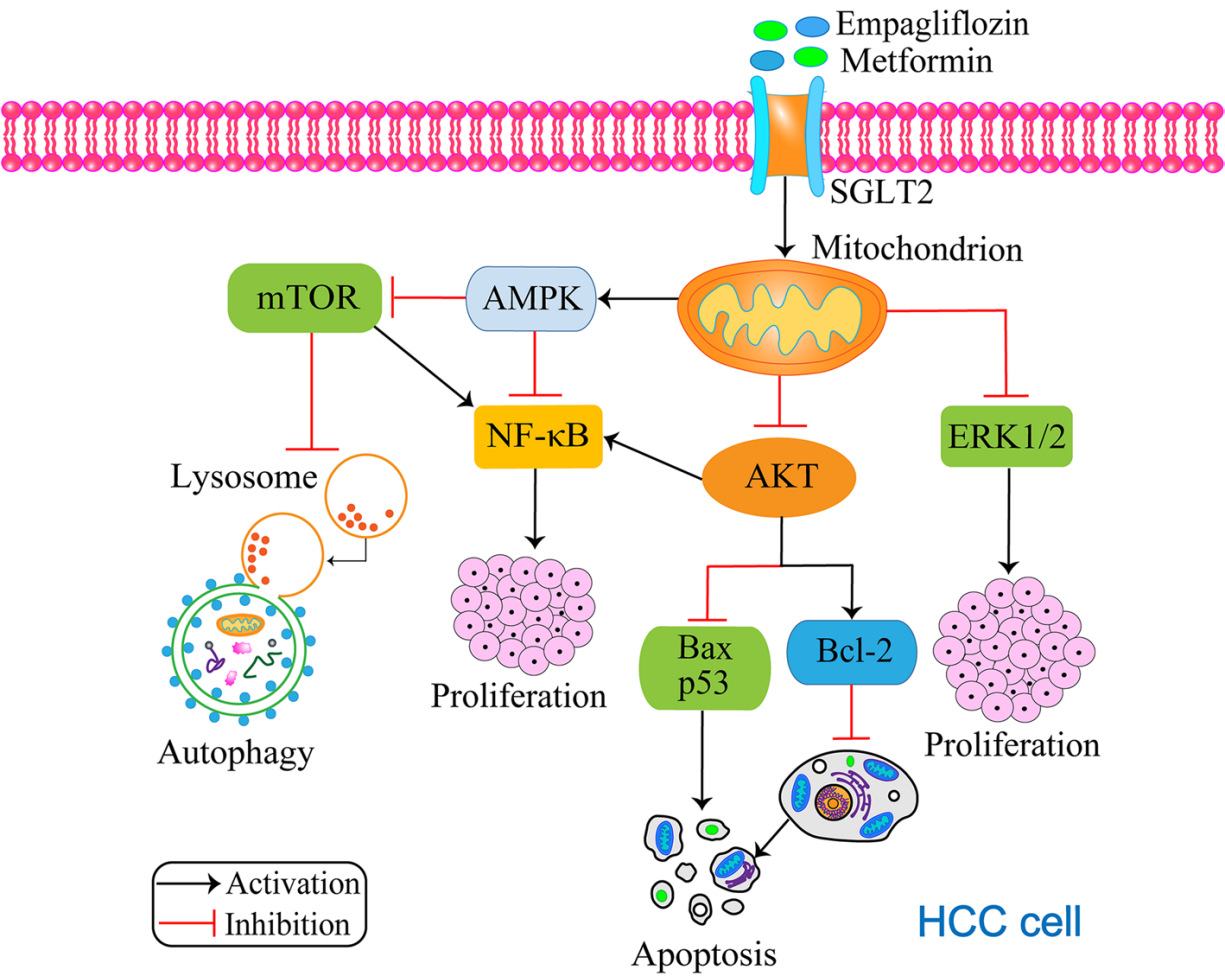
The combination of empagliflozin and metformin inhibits progression of hepatocellular carcinoma. Both drugs activate AMPK, leading to inhibition of *mTOR* expression, NF-κB inactivation, and ultimately, reduction of cell proliferation. Another potential mechanism involves the combination’s ability to inhibit AKT, also resulting in NF-κB inactivation. This inhibition of AKT also decreases *Bcl-2* expression while upregulating Bax and p53, thus leading to apoptosis

Hepatocellular carcinoma (HCC) is a high-mortality cancer, and its incidence is increasing worldwide. Infection with hepatitis B virus (HBV) or hepatitis C virus (HCV) is one of the most important causes of HCC, chronic liver disease formed by long-term alcohol abuse or metabolic disorders can also cause HCC [58], and the incidence of HCC is 2–3 times higher in patients with diabetes than in normal subjects [59]. One study reported that the combination of the antidiabetic drugs empagliflozin and metformin had some degree of inhibitory effect on the proliferation of HCC cells [19]. Empagliflozin treatment prevents liver fibrosis as well as hepatic steatosis [60], and metformin reduces protein levels of the cancer marker CD133 through the AMPK-CCAAT/enhancer-binding protein beta (CEBPβ) signaling pathway [61]. Empagliflozin increases the antitumor effect of metformin to some extent when empagliflozin and metformin are combined to treat HCC. By inducing HCC formation in mice with diethylnitrosamine (DEN), Abdelhamid et al. [19] found that the proliferation of HCC cells was inhibited and survival was prolonged after treatment with empagliflozin adjuvanted with metformin (Fig. 3). This may be due to the activation of AMPK by metformin, which causes nuclear factor kappa-light-chain-enhancer of activated B cells (NF-κB) signaling molecules to be inhibited. NF-κB is a key transcriptional regulator of the inflammatory response, and NF-κB activation has been detected in almost all patients with chronic liver disease, and patients with HCC are no exception [62]. Empagliflozin did not exhibit the ability to inactivate NF-κB signaling molecules, but it had the ability to inhibit p38 MAPK and ERK1/2 activity [19]. The MAPK signaling pathway is involved in oxidative stress [63], inflammatory responses [64], and cell proliferation [65], a finding that confirms the study of Lin et al. [66]. The results of this study suggest that the inhibitory effect of columbamine (Col) on HCC is associated with the p38 MAPK and ERK1/2 signaling pathways [66]. In this study, both empagliflozin and metformin were found to activate AMPK, which resulted in the inhibition of *mTOR* expression, thereby leading to NF-κB inactivation and suppression of HCC growth. Another potential mechanism involves the combined use of empagliflozin and metformin, which inhibits AKT and subsequently inactivates NF-κB [19]. This AKT inhibition leads to a decrease in *Bcl-2* expression and an upregulation of *Bax* and *p53* expression, resulting in apoptosis. Therefore, empagliflozin in combination with metformin is expected to be a new potential chemotherapy combination for the treatment of HCC.

### **Empagliflozin and lung cancer**

Lung cancer is the most common type of cancer in China and the leading cause of cancer deaths worldwide [67]. Smoking is an important factor in causing lung cancer [68]. Implementation of screening programs for high-risk groups as well as early diagnosis is one of the main ways to reduce mortality in patients with lung cancer [69]. Faridi et al. [2] studied the effect of empagliflozin on the human-derived lung cancer cell Line A549, and the experimental results showed that empagliflozin had a significant inhibitory effect on A549 cells. In addition, the researchers also conducted a computer simulation study of the anticancer targets of empagliflozin through molecular docking software and initially identified the apoptosis-related proteins Bcl-2, p53 and Caspase-3 [2]. The 3D structures of proteins were obtained from the Protein Database (PDB), and the 3D structure of empagliflozin was obtained from PubChem [2]. The 3D structures of empagliflozin and three apoptotic proteins were docked by molecular docking software. The study revealed that the apoptotic protein receptors 1GJH, 1TUP, and 2XYG and empagliflozin ligand had − 348.12, -300.12, and 203.36 E values, respectively, indicating that they were likely to bind [2]. Moreover, treatment of A549 cells with empagliflozin at a concentration of 100 µg/ml resulted in only 51.49% cell viability, indicating that the IC_50_ concentration of empagliflozin inhibiting A549 cells was approximately 110 µg/ml. Interestingly, the IC_50_ concentration of glimepiride, a diabetic drug, inhibiting A549 cells was nearly twice as high at approximately 240 µg/ml [2]. These findings highlight the potential of empagliflozin as an effective inhibitor of lung cancer cells. In addition, Sinha et al. [70] prepared an orally dispersible film agent with chitosan-sodium alginate nanoparticles loaded with empagliflozin. Studies demonstrated that empagliflozin chitosan-sodium alginate nanoparticles had a relatively high bioavailability in the Wistar rat model and were 2.5 times more cytotoxic to A549 lung cancer cells than free empagliflozin [70]. Treatment of A549 lung cancer cells with the oral dispersion film agent of chitosan-sodium alginate nanoparticles loaded with empagliflozin resulted in a significantly lower IC_50_ concentration of 69.24 µg/ml compared to treatment with free empagliflozin, suggesting the potential of this dispersion film agent as a more effective treatment option [70]. The mechanism of action of empagliflozin in treating lung cancer remains unclear, but it has been shown that SGLT2 is a target of action in several cancers as well as diabetes [71]. Inhibition of normal glycolysis in tumor cells through targeting SGLT2 may provide a starting point for exploring the potential of empagliflozin as a dual-use drug for the treatment of patients with diabetes and cancer, but further research is needed to provide more detailed data to support this potential use of empagliflozin.

## Summary and prospects

Empagliflozin, an SGLT2 inhibitor, is effective in the treatment of T2D. It also prevents hypoglycemia and helps patients control their weight. The anti-inflammatory effect of empagliflozin may help diabetic patients alleviate the complications caused by diabetes. Empagliflozin exhibits cardioprotective effects, making empagliflozin potentially useful for the treatment of cardiovascular disease in patients with heart failure. In addition, empagliflozin exhibits anticancer activity in some types of cancer, including inhibition of tumor cell proliferation, migration, invasion, and induction of apoptosis. When used in combination with certain chemotherapeutic agents or radiation therapy, empagliflozin may improve therapeutic efficacy while reducing associated side effects. The possible mechanisms of SGLT2 inhibitors’ actions were summarized by Dutka et al. [20]. These mechanisms include inhibition of β-linked proteins, activation of the AMPK signaling pathway, cell cycle arrest, and inhibition of EGFR [20]. Canagliflozin and dapagliflozin were found to activate AMPK, which led to inhibition of mTOR in breast cancer. Additionally, canagliflozin was observed to inhibit the expression of *SREBP1* and *SCD1* in breast cancer cells and HCC cells [20]. Empagliflozin was also found to activate AMPK, which induced apoptosis in cervical cancer cells [17]. These findings suggest that AMPK could be a potential target for gliflozin-like drugs in cancer treatment. Canagliflozin was also observed to inhibit HCC cell cycle arrest via the inhibition of phosphorylation of ERK1/2 and p38 [20]. Additionally, empagliflozin was found to inhibit the development and progression of HCC by inhibiting p38 MAPK and ERK1/2 [19]. Thus, it can be speculated that there exist similarities in the mechanisms of action of gliflozin-like drugs. Despite the in-depth summary of canagliflozin and dapagliflozin in the treatment of various cancers, empagliflozin’s mechanisms of action were only mentioned in two cancer types. This review takes empagliflozin as an anticancer drug and summarizes its therapeutic effects on several cancers based on existing studies. This information provides a reference for future research on empagliflozin’s use as a chemotherapy drug or an adjuvant to chemotherapy drugs while hoping to develop its potential in cancer treatment.
